# The Use of Grape Seed Byproducts Rich in Flavonoids to Improve the Antioxidant Potential of Red Wines

**DOI:** 10.3390/molecules21111526

**Published:** 2016-11-12

**Authors:** María José Jara-Palacios, Dolores Hernanz, María Luisa Escudero-Gilete, Francisco J. Heredia

**Affiliations:** 1 Food Colour & Quality Laboratory, Department of Nutrition & Food Science, Facultad de Farmacia, Universidad de Sevilla, 41012 Sevilla, Spain; mjara@us.es (M.J.J.-P.); gilete@us.es (M.L.E.-G.); 2 Department of Analytical Chemistry, Facultad de Farmacia, Universidad de Sevilla, 41012 Sevilla, Spain; vila@us.es

**Keywords:** seeds, grape pomace, flavonoids, antioxidant activity, red wine

## Abstract

The influence of adding seeds from grape pomace during Syrah wine fermentation in a warm climate has been studied. Seeds of Pedro Ximenez variety were rich in phenolic compounds, mainly flavonoids such as catechin and procyanidins. Changes in total phenolic content (TPC), total flavonoid content (TFC), and antioxidant activity of red wines were observed. These changes depended on the vinification stage and the amount of seeds (SW: 450 g or DW: 900 g seeds/150 kg grapes) applied. In general, antioxidant activity was greater when a simple dose (SW) was considered. Results indicate that seeds rich in flavonoids could be used as wine additives, which could improve the antioxidant potential of red wines in a warm climate.

## 1. Introduction

Flavonoids are phenolic compounds widely distributed in the plant kingdom, and they are very important determinants in the nutritional and sensory quality of fruits and vegetables. Flavonoids are low molecular weight compounds and can be classified into six major classes: anthocyanins, flavones, isoflavones, flavanones, flavonols, and flavanols [1]. These compounds are very abundant in fruits and vegetables such as grapes, apples, blueberries, onions, and lettuce and plant-derived liquids such as tea, wine, and cacao [2,3,4].

Flavonoids have received attention because of their biological/pharmacological activities that are related to the prevention of common diseases, such as cancer, neurodegenerative diseases, cardiovascular, and gastrointestinal disorders [5,6,7,8], and to the fight against ageing [9]. These beneficial effects of flavonoids on health have been associated with their antioxidant activity and ability to decrease the oxidative stress, which is related to the physiopathology of many diseases [10].

Nowadays, consumers are more conscious of the importance of a healthy and good diet that includes natural antioxidants with influence on the health. In this sense, Mediterranean diet is one of the healthier diet models [11], and adherence to it is associated with a good lifestyle [12]. It is rich in fruits, vegetables, nuts, fish, and olive oil, as well as in red wine, which is an abundant source of anthocyanins, flavanols, flavonols and other phenolic compounds such as phenolic acids [13,14,15]. Studies have suggested that the consumption of wine has beneficial effects on cardiovascular health, which is in part due to alcohol content (10%–15%), because moderate consumption increases HDL [16]. In addition, wine possesses a large variety of beneficial effects against human diseases due to the protective effect of phenolic compounds [17,18,19].

The current interest in the benefits of moderate wine drinking on health has promoted that much of the food industry is actively involved in the functional food development of wine. Functional foods are foods that are intended to be consumed as part of a normal diet and that contain biologically active components that offer the potential of enhanced health or a reduced risk of disease. Wine would appear to fit this definition; however, while natural improvements of phenolic content and antioxidant activity are desirable, the addition of compounds into wine may be required for the promotion of wine as a functional food [20]. For example, the addition of plant extracts could increase the health benefits in wine; however, some phenolic compounds in these could alter the flavor of wine. For this reason, consumer preference is a very important factor in supplementation. Previous studies have reported that consumer preference varies depending on cultures, geographical, and socially diverse areas [21,22]. A previous study of wines spiked with green tea or grape seed extracts determined consumer rejection thresholds in different cultural groups, which affected preference [21]. On the other hand, different pathways are necessary to wine being accepted as a functional food: functionality, price, taste, moderate drinking, and consumer perception of the healthiness of wine [16]. Saliba et al. [23] studied consumer perception and about one-quarter of wine drinkers decided that wine was healthy. This perception depended on factors such as country, age, and gender.

Spain, one of the largest producers of wine in the world (36.6 mhl) after Italy (48.9 mhl) and France (47.4 mhl), is a geographical area with typical climatological conditions of warm climate, where stressful climate conditions make it difficult to obtain high quality red wines, mainly affecting color [24]. In some regions of this country, this is a persistent issue since phenolic maturity does not coincide with the technological (sugars) maturity of grapes; therefore, at the moment of harvesting, different levels of both phenolic and sugar maturity exist. Thus, copigmentation is affected in the first steps of the winemaking process; as a result, color stabilization does not correctly develop [25,26,27,28]. 

In these warm regions, an extra contribution of phenolic compounds could be necessary to solve the problem. It is necessary to use a source of phenolic compounds that do not affect a wine’s astringency; therefore, we propose the addition of ripe-seeds. The stressful climate conditions could also affect the antioxidant activity of wines, and the addition of seeds could improve the biological properties of wines.

The external addition of phenolic compounds from natural sources to the musts prior to, during, or after fermentation has been reported for the purpose of color stabilization and phenolic composition improvement [25,29,30,31,32]; however, in these works, the antioxidant potential of wine was not studied.

Winemaking byproducts, such as grape pomace, contain large amounts of phenolic compounds with antioxidant properties and benefits on human health [33]. Seeds, skins, and stems from grape pomace exhibit different phenolic profiles. Seeds have the highest total phenolic content, being flavanols the most abundant compounds [34]. Therefore, grape seeds are a natural source of phenolic compounds, particularly flavanols, which could be added during wine fermentation in order to improve the antioxidant activity of wines. 

The aim of this work was to study the effect of adding grape seeds at different concentrations on the antioxidant potential of Syrah wines in a warm climate. 

## 2. Results and Discussion

### 2.1. Phenolic and Antioxidant Characterization of Grape Seeds

First, the seeds used during winemaking were analyzed in order to determine its phenolic composition and antioxidant potential. The total phenolic content (TPC), individual phenolic compounds, and antioxidant activity of the seeds are shown in Table 1. The TPC evaluated by the Folin–Ciocalteu assay was 5535 mg/100 g of dry matter (DM). A total of 19 phenolic compounds (flavonoids and non-flavanoids) were identified and quantified via UHPLC: 14 flavanols, 2 benzoic acids, and 3 hydroxycinnamic acids. Flavanols were the major compounds detected, accounting for 90% of total phenolic compounds (706 mg/100 g of DM), indicating that considerable amounts of flavonoids can be recovered from Pedro Ximénez winemaking byproducts. As can be seen in Table 1, two monomeric flavanols were the most abundant flavonoids (catechin and epicatechin), followed by two oligomeric flavanols (procyanidins B2-3-*O*-gallate and B1). Gallic acid, a non-flavonoid compound, was the main phenolic acid found in the seeds (36 mg/100 g of DM). These phenolic compounds have previously been identified in grape pomace from Pedro Ximénez variety although in different concentrations [29,35].

The phenolic compounds present in the seeds have critical importance because it is well-known that they have antioxidant activity [36,37,38]. Thus, the antioxidant activity of seed extracts was measured with ABTS and FRAP assays (Table 1). Seeds showed antiradical and ferric reducing antioxidant powers (48 and 23 mmol TE/100 g of DM, respectively). Previous studies have reported that winemaking byproducts from Pedro Ximénez and other grape varieties have great antioxidant activity [34,35]. 

### 2.2. Phenolic Profile of Wines and Evolution

Table 2 summarizes the total phenolic content and the total flavonoid content (TFC) of control Syrah wines (CW) and wines treated with the addition of seeds (simple and double dose; SW and DW, respectively) at different points of the vinification process: the initial point (I), at the middle of the fermentative alcoholic (FAM), at the end of malolactic fermentation (MLF), and at the end of treatment (4 months of stabilization: W1, W2, W3, and W4). 

At the initial point, the addition of the seeds did not result in a higher TPC (2380, 2279, and 2282 mg/L for CW, SW, and DW, respectively; *p* > 0.05) or TFC, whose values were significantly lower (1074, 870, and 867 mg/L for CW, SW, and DW, respectively; *p* < 0.05). However, at stage FAM, the values of TPC and TFC for wines containing a simple dose of seeds were higher than for control wines, although a significant difference was not found. At stage MLF, the TPC and TFC for wines containing a simple and double dose of seeds were lower than for control wines (Table 2). These lower values could be due to the saturation of the medium, compound sedimentation, or the partial adsorption of certain phenolic compounds (such as proanthocyanidins) by the cell wall material [39,40,41]. As can be observed in Table 2, the TPC and TFC started to increase for SW and DW at the months of stabilization, and they generally had higher values than CW. Therefore, after 3 and 4 months of stabilization (W3 and W4, respectively), an increase in TPC and TFC resulted from adding seeds, and the effect was greater when a simple dose was considered. Previous studies have indicated that the effect on phenolic content depends on the proportions of seeds applied because a proportion that is too high may damage the wine quality [29,30]. These studies have also indicated that the addition of phenolic compounds from natural sources represents an interesting and innocuous enological practice to improve phenolic potential, which improves sensory properties such as color stability [26]. The proportion of seeds applied is also very important because high phenolic content could increase the astringency of the wine [42]. Results showed that addition before or during the fermentative step of the winemaking did not adversely affect fermentation. We did not observe negative effects in fermentation due to the addition of seeds.

### 2.3. Antioxidant Activity of Wines and Evolution

**Figure 1:**
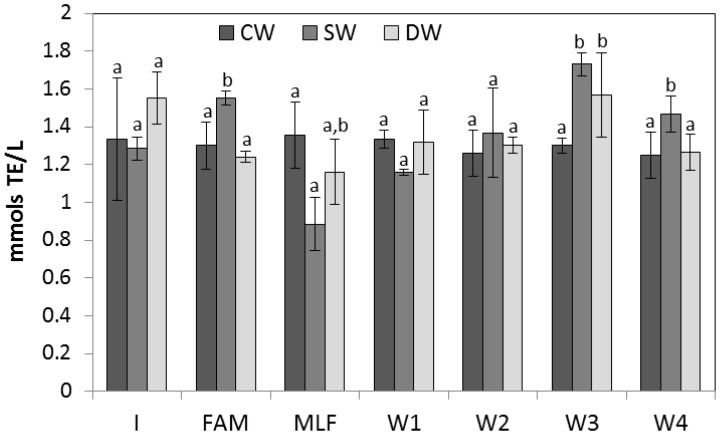
Values of antioxidant activity measured with the ABTS method during the winemaking process in CW, SW, and DW. Different letters in the same stages of winemaking indicate significant differences between CW, SW, and DW by ANOVA test (*p* < 0.05).

**Figure 2:**
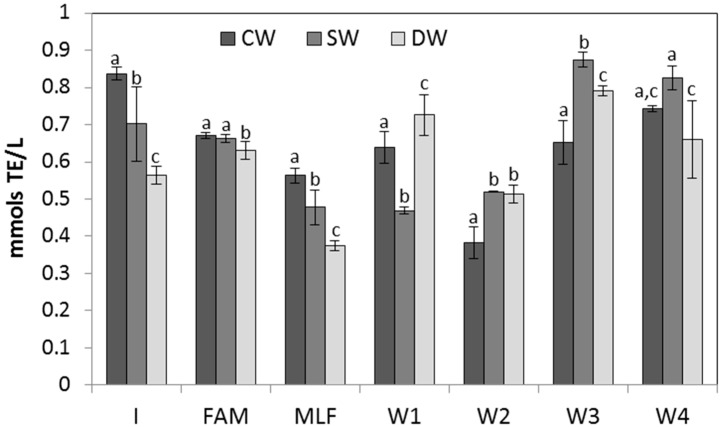
Values of antioxidant activity measured with the FRAP method during the winemaking process in CW, SW, and DW. Different letters in the same stages of winemaking indicate significant differences between CW, SW, and DW by ANOVA test (*p* < 0.05).

The evolution of antioxidant activity (measured by ABTS and FRAP methods) of wines during the winemaking processes is shown in Figure 1 and Figure 2. Results showed that the evolution of antioxidant activity, in general terms, was in accordance with the evolution of TPC and TFC, albeit with some variations. The antioxidant activity differed depending on the type of wine (CW, SW, or DW), the winemaking stage, or the stabilization time. 

Regarding the ABTS method, values for CW remained stable during all stages (from 1.33 to 1.25 mmol/L). The antioxidant activity for SW and DW was variable during the process, showing a decrease at MLF (42% and 7% less than at FAM, respectively), which could be due to a lower concentration of flavonoids at this stage. During stabilization time, the antioxidant activity levels for SW and DW increased, and they were higher than those for CW after 2, 3, and 4 months of stabilization (Figure 1). The greatest value was found for SW after 3 months of stabilization (1.73 mmol/L) followed by DW (1.57 mmol/L), with 25% and 17% more than value of CW (1.30 mmol/L). 

Respect to the results from the FRAP method, the general evolution of antioxidant activity is similar to the ABTS method, although some differences were found. Values for CW, SW, and DW fluctuated during the entire process. In the first stages, the values of CW were more elevated than those of SW and DW; however, at stabilization time, the opposite occurred (Figure 2). Greater antioxidant activity was found at stabilization time with significantly (*p* < 0.05) higher values for SW and DW at 2, 3, and 4 months of stabilization, showing the highest value for SW at 3 months (0.87 mmol/L). 

This positive trend in antioxidant activity for SW at 3 and 4 months of stabilization could be related to the higher levels of phenolic compounds, mainly flavonoids, which can proceed from seeds.

**Figure 3:**
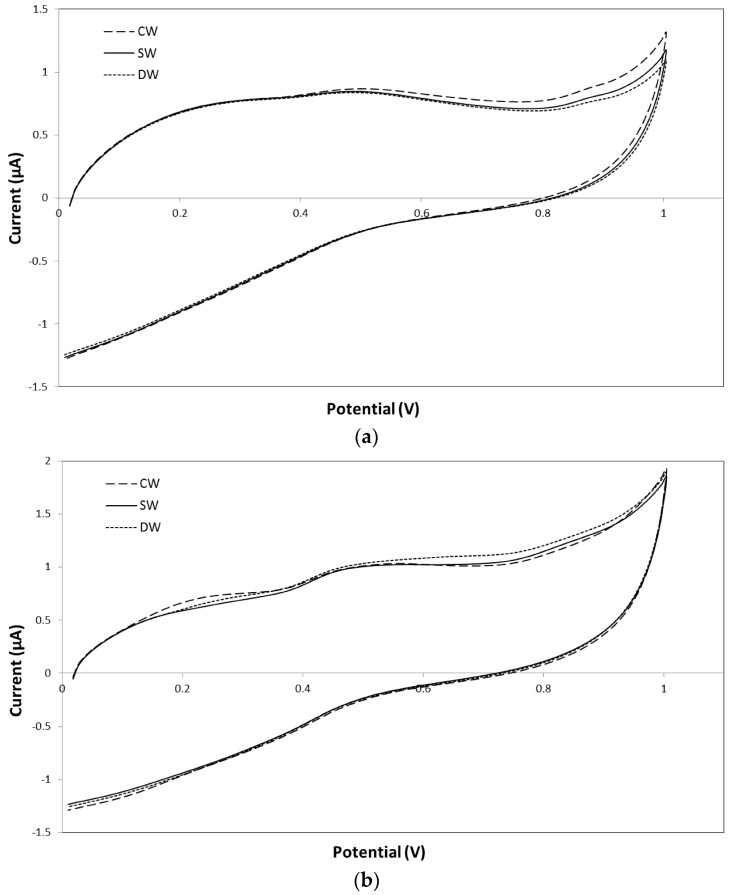
Cyclic voltammograms of CW, SW, and DW at different stages: (**a**) stage I; (**b**) stage W4.

Finally, cyclic voltammetry (CV) was used to determinate the electrochemical behavior of the wines at the beginning of treatment and at the end of treatment in order to study their antioxidant activity. Table 3 shows the electrochemical parameters corresponding to the area under the curve (QT, QI, QII, and QIII) extracted from the cyclic voltammetry curves of the wines. Figure 3 shows the cyclic voltammograms of 25-fold diluted wines, at stage I (a) and stage W4 (b), for scan from 0 to 1 V.

Regarding QT, CW had higher values at stage I than SW and DW samples (0.740 versus 0.708 and 0.693, respectively). The area under the curve can be related to the concentration of total phenols [43]; therefore, these differences could be due to higher TPC and TFC at this stage. On the contrary, at the end of the stabilization period (W4), the lowest value was found for SW (0.887) and the highest for DW (0.925). At this stage, with respect to QII, DW had higher values (0.344) than CW (0.330) and SW (0.328); values of QIII were higher for DW (0.405) and SW (0.389) than for CW (0.386). The area under the curve of the third interval (QIII) is generally due to the second oxidation step of flavonoids or phenolic acids, although is often difficult to analyze and correlate to the phenolic profile [44,45]. Electrochemical results indicate that DW samples had better antioxidant activities than CW, which do not coincide with the results of the ABTS and FRAP methods. This discordance could be due to the fact that there is no linear correlation between CV parameters and the results of the ABTS assay and that CV technique measures the global antioxidant activity and not only the activity due to the phenolic contents [35,45].

## 3. Experimental Section

### 3.1. Chemical and Reagents

Hydrochloric acid, formic acid, HPLC-grade acetonitrile, methanol, ethanol, glycine, Folin-Ciocalteu reagent, and iron trichloride (FeCl_3_·6H_2_O) were obtained from Panreac (Barcelona, Spain). ABTS (2,2-azino-bis-(3-ethylbenzothiazolne-6-sulfonic acid) diammonium salt) and Trolox (6-hydroxy-2,5,7,8-tetramethyl-chroman-2-carboxylic acid) were purchased from Fluka (Madrid, Spain).

Gallic acid, protocatechuic acid, (+)-catechin, (−)-epicatechin, caffeic acid, *p*-coumaric acid, sodium carbonate, potassium persulphate, 2,4,6-tris(2-pyridyl)-s-triazine (TPTZ), and phosphate buffered saline (PBS) were purchased from Sigma-Aldrich (Madrid, Spain). 

### 3.2. Samples and Winemaking

Grapes from *Vitis vinifera* cv. Syrah (red grape) cultivated in ‘‘Condado de Huelva” D.O. in Southwestern Spain (warm climate) and seeds from *Vitis vinifera* cv. Pedro Ximenez (white grape) grown in “Montilla-Moriles” D.O. were used in this study. 

Grape pomace was provided by “La Aurora” winery (Montilla, Córdoba, Spain) after Pedro Ximénez grapes were processed for winemaking at an overripe stage (24 °Baumé). Grapes had been previously sundried for 12 days using esparto grass mats. The grape pomace contained only some stems that were manually separated. Seeds were separated from skins using a suitable sieve and were frozen until 24 h before winemaking.

Approximately 1350 kg of Syrah grapes were manually harvested in “Condado de Huelva” at optimum technological maturity (12.4 °Baumé) and sanitary conditions. The grapes were destemmed and crushed, and the must with solid parts was homogenized and placed in stainless steel tanks (220 L each) for skin maceration. 

A total of nine vinifications were performed. Three types of experimental vinifications, in three replicates for each one (*n* = 3), were performed with mixtures of Syrah grapes and Pedro Ximenez seeds as follows: (1) wines elaborated with 150 kg of Syrah grapes were used as the control (CW); (2) wines containing 150 kg of Syrah grapes and 450 g of seeds constituted the simple dose (SW); (3) wines containing 150 kg of Syrah grapes and 900 g of seeds constituted the double dose (DW). 

All experiments were carried out following an identical red winemaking procedure as previously described [30]. Wines were adjusted at the same levels: 60 mg/L total sulfur dioxide and 7 g/L of total titratable acidity was achieved by adding tartaric acid. Fermentative alcoholic maceration was induced by inoculating *Saccharomyces cerevisiae* selected yeast; then, at the end of alcoholic fermentation, selected *Oenococcus oeni* lactic acid bacteria were inoculated at a rate of 10 mL/hL. 

Wine samples (50 mL) were taken at different stages: at the initial point or skin removal (I), at the middle of the fermentative alcoholic (FAM), at the end of malolactic fermentation (MLF), and 1, 2, 3, and 4 months after fermentative processes were finished (stabilization time; W1, W2, W3, and W4, respectively).

### 3.3. Extraction of Phenolic Compounds from Seeds

Pedro Ximenez seed samples were frozen at 20 °C, freeze-dried for 24 h, and finally ground to a fine powder. The freeze-dried seeds (1 g) were extracted, in triplicate, with 75% methanol as an extraction solvent following the method described [6]. The resulting phenolic extracts were frozen until analysis.

### 3.4. Determination of Total Phenolic Content

Total phenolic content (TPC) was determined using the Folin–Ciocalteu method with some modifications [34]. Absorbance was measured at 765 nm, and the results were expressed as milligrams of gallic acid per liter (mg GAE/L) for wines and as milligrams of gallic acid per 100 g (mg GAE/100 g) for seeds.

### 3.5. Determination of Total Flavonoids

Total flavonoid content (TFC) was determined by the aluminum chloride colorimetric method as previously described [46]. Wine samples (0.5 mL) were mixed with 2 mL of distilled water and 150 µL of a 5% NaNO_2_ solution. After 5 min, 150 µL of 10% AlCl_3_ were added and, after 6 min, 2 mL of a 1 mol/L NaOH solution were also added. The final volume was brought to 5 mL with distilled water. Finally, the absorbance was measured at 510 nm. Results were expressed as mg catechin equivalent per liter (mg CE/L).

### 3.6. UHPLC-DAD Analysis of Phenolic Compounds

An Agilent 1290 chromatograph (Agilent Technologies, Palo Alto, CA, USA), equipped with a diode-array detector and an Eclipse Plus C18 Agilent column (1.8 µm, 2.1 × 50 mm) at 25 °C, was used to the chromatographic analysis. The flow rate was 1 mL/min, and the solvents were 0.1% formic acid in water (solvent A) and acetonitrile (solvent B) at the following gradient: 0–5 min, 5% B linear; 5–20 min 50% B linear; 20–22 min, washing and re-equilibration of the column.

The identification and quantification of phenolic compounds was carried out as previously described [34].

### 3.7. ABTS/Persulfate Assay

The ABTS assay was performed as previously described [47]. Extract or wine (50 µL) was added to 2 mL of the ABTS^•+^ diluted solution (7 mM ABTS with 2.45 mM potassium persulfate in water). The absorbance was measured at 734 nm after incubation at room temperature for 4 min, and results were expressed as Trolox equivalent antioxidant capacity (TEAC), considered as the mmol of Trolox with the same antioxidant capacity as 1 L of wine (mmol TE/L) or 100 g of seeds (mmol TE/100 g).

### 3.8. FRAP Assay

The FRAP assay was performed as described previously [48]. Extract or wine (100 µL) was added to 3 mL of the FRAP reagent (10 mM TPTZ in 40 mM·HCl, 20 mM·FeCl_3_, and 300 mM acetate buffer at pH 3.6 (proportion 1:1:10, *v*/*v*/*v*)). The absorbance was measured at 593 nm after incubation at room temperature for 6 min, and results were expressed as Trolox equivalent antioxidant capacity (TEAC), considered as the mmol of Trolox with the same antioxidant capacity as 1 L of wine (mmol TE/L) or 100 g of seeds (mmol TE/100 g).

### 3.9. Cyclic Voltammetry

Electrochemical measurements were realized by using a potentiostat/galvanostat (AUTOLAB model PGSTAT 302 N) controlled by a General Purpose Electrochemical System (GPES) software (MetrohmAutolab B.V., Utrecht, The Netherlands). 

Analyses were performed following the method proposed by Jara-Palacios et al. 2014 [35], with some modifications. Wine (1 mL) was diluted with a 0.1 M sodium acetate-acetic acid buffer at pH 3.6 and transferred into a glass water-jacketed electrochemical cell connected to a circulator (temperature = 25.0 ± 0.5 °C). Prior to the measurements, the electrolyte solutions were de-aerated with an inert gas (N_2_) for 5 min. All measurements were carried using a conventional three-electrode system: a glassy carbon working electrode, a platinum auxiliary electrode, and a Ag/AgCl reference electrode. The cyclic voltammogram scans were recorded in triplicate from 0.0 to 1.0 V at a scanning rate of 5 mV/s.

The electrochemical parameter extracted from the cyclic voltammetry curves was the anodic current area (QT), which represents the total integrated area of the cyclic voltammogram for scans taken from 0 to 1 V. In addition, the area was measured at three intervals: QI represents the integrated area from 0 to 0.35 V, QII from 0.35 to 0.70 V, and QIII from 0.70 to 1 V. 

### 3.10. Statistical Analysis

The Statistica v.8.0 software was used for the statistical treatment of the data. One-way analysis of variance (ANOVA) was employed to establish if TPC, TFC, and antioxidant activity differed significantly between CW, SW, and DW in the different stages of winemaking. In all cases, *p* < 0.05 was considered statistically significant.

## 4. Conclusions

In summary, seeds obtained from grape pomace of the Pedro Ximénez variety are a rich source of flavonoids such as flavanols with antioxidant properties. Results indicate that the addition of grape seeds offers a promising technique for increasing the flavonoid content and consequently the antioxidant activity in red wines in a warm climate. This effect depends on the amount of seeds applied; in general terms, a simple dose was greater than a double dose of seeds. Nevertheless, further studies are required to elucidate the components (flavanols or other compounds present in seeds, such as phenolic acids), which are responsible for these effects. In addition, consumer rejection thresholds and preference studies should be stablished for these wines in order to determine the acceptance of these wines as a healthy and functional food. 

This study could be of great interest in the wine and food industry because the proposed technique will allow wine producers to improve the antioxidant potential of wines, which is very important for actual consumers.

## Figures and Tables

**Table 1:** Concentrations of phenolic compounds in seeds from grape pomace of the Pedro Ximénez variety.

Phenolic Compounds	Concentration (mg/100 g)
Benzoic acids	
Gallic acid	35.84 ± 0.07
Protocatechuic acid	9.25 ± 0.07
Hydroxycinnamic acids	
Caffeic acid	7.60 ± 0.01
p-Coumaric acid	10.00 ± 0.03
t-Coutaric acid	10.59 ± 0.02
Flavanols	
Catechin	87.59 ± 0.72
Epicatechin	85.04 ± 2.31
Epicatechin gallate	48.90 ± 0.35
Procyanidin B1	74.62 ± 0.21
Procyanidin B2	61.54 ± 1.05
Procyanidin B3	31.50 ± 0.63
Procyanidin B4	26.03 ± 0.08
Procyanidin trimer 1	27.98 ± 0.06
Procyanidin trimer 2	38.09 ± 0.02
Procyanidin tetramer 1	27.43 ± 0.29
Procyanidin tetramer 1	41.78 ± 0.06
Procyanidin B2-3-*O*-gallate	78.35 ± 1.26
Galloyled procyanidin 1	30.46 ± 0.21
Galloyled procyanidin 2	45.07 ± 0.42
ΣPhenols	780.64 ± 0.74
TPC (mg/100 g)	5534.67 ± 17.80
ABTS (mmol TE/100 g)	48.24 ± 1.40
FRAP (mmol TE/100 g)	22.69 ± 3.12

ΣPhenols: sum of all of individual phenolic compounds.

**Table 2:** Total phenolic content (TPC) and total flavonoid content (TFC) in red wines, at the different stages of winemaking.

	Stage	CW	SW	DW
TPC	I	2379.72 ^a^ ± 109.95	2279.07 ^a^ ± 116.51	2281.97 ^a^ ± 46.81
	FAM	2046.72 ^a^ ± 52.18	2221.79 ^a^ ± 150.75	2083.11 ^a^ ± 80.42
	MLF	2073.42 ^a^ ± 104.95	1928.82 ^a^ ± 40.71	1996.15 ^a^ ± 200.05
	W1	2230.32 ^a^ ± 107.68	2280.46 ^a^ ± 230.09	2299.98 ^a^ ± 329.48
	W2	2313.81 ^a^ ± 62.51	2387.15 ^a^ ± 31.66	2274.15 ^a^ ± 175.65
	W3	2457.68 ^a^ ± 245.46	2833.98 ^b^ ± 212.47	2472.35 ^a^ ± 257.45
	W4	2291.38 ^a^ ± 152.81	2344.54 ^a^ ± 88.37	2320.39 ^a^ ± 64.19
TFC	I	1074.20 ^a^ ± 30.30	870.16 ^b^ ± 3.54	867.39 ^b^ ± 3.59
	FAM	941.23 ^a^ ± 87.57	968.44 ^a^ ± 36.78	706.25 ^b^ ± 49.94
	MLF	1004.12 ^a^ ± 88.16	743.88 ^b^ ± 49.35	848.56 ^b^ ± 46.14
	W1	955.87 ^a^ ± 20.13	924.51 ^a^ ± 49.43	939.33 ^a^ ± 23.89
	W2	1032.73 ^a^ ± 47.19	934.43 ^b^ ± 33.82	1027.73 ^a^ ± 63.03
	W3	1036.24 ^a^ ± 18.13	1152.88 ^b^ ± 95.23	923.51 ^c^ ± 1.68
	W4	1140.15 ^a^ ± 34.95	1192.96 ^a^ ± 46.01	1046.38 ^b^ ± 64.40

^a,b,c^: Values in the same row followed by different letters are significantly different by ANOVA test (*p* < 0.05).

**Table 3:** Values of the area under the curve (QT, QI, QII, and QIII) extracted from the cyclic voltammetry curves of the red wines at stage I and W4.

Stage		CW	SW	DW
I	QT	0.740	0.708	0.693
	QI	0.191	0.191	0.189
	QII	0.282	0.274	0.271
	QIII	0.267	0.243	0.233
W4	QT	0.900	0.887	0.925
	QI	0.184	0.170	0.175
	QII	0.330	0.328	0.344
	QIII	0.386	0.389	0.405
